# Metabolomic Biomarkers in Anxiety Disorders

**DOI:** 10.3390/ijms21134784

**Published:** 2020-07-06

**Authors:** Elke Humer, Christoph Pieh, Thomas Probst

**Affiliations:** Department for Psychotherapy and Biopsychosocial Health, Danube University Krems, 3500 Krems, Austria; christoph.pieh@donau-uni.ac.at (C.P.); thomas.probst@donau-uni.ac.at (T.P.)

**Keywords:** metabolomics, metabolites, anxiety, biomarkers

## Abstract

Anxiety disorders range among the most prevalent psychiatric disorders and belong to the leading disorders in the study of the total global burden of disease. Anxiety disorders are complex conditions, with not fully understood etiological mechanisms. Numerous factors, including psychological, genetic, biological, and chemical factors, are thought to be involved in their etiology. Although the diagnosis of anxiety disorders is constantly evolving, diagnostic manuals rely on symptom lists, not on objective biomarkers and treatment effects are small to moderate. The underlying biological factors that drive anxiety disorders may be better suited to serve as biomarkers for guiding personalized medicine, as they are objective and can be measured externally. Therefore, the incorporation of novel biomarkers into current clinical methods might help to generate a classification system for anxiety disorders that can be linked to the underlying dysfunctional pathways. The study of metabolites (metabolomics) in a large-scale manner shows potential for disease diagnosis, for stratification of patients in a heterogeneous patient population, for monitoring therapeutic efficacy and disease progression, and for defining therapeutic targets. All of these are important properties for anxiety disorders, which is a multifactorial condition not involving a single-gene mutation. This review summarizes recent investigations on metabolomics studies in anxiety disorders.

## 1. Introduction

Anxiety disorders are a prevalent global health problem, affecting the lives of almost 300 million individuals suffering from a range of anxiety disorders as well as society as a whole [1]. Anxiety disorders are currently the most prevalent psychiatric disorder in the United States and Europe and are ranked by the WHO as the sixth largest cause of disability worldwide and range among the top ten causes of years lived with disability [1,2]. Anxiety disorders also lead to the subsequent development of other psychiatric comorbidities, such as depression [3]. The prevalence of anxiety disorders is affected by gender, with a higher prevalence in women than men [4]. Despite a trend towards lower prevalence among older people (≥80 years), prevalence rates are similar among age groups [1,5]. The group of anxiety disorders is characterized by feelings of anxiety and fear and related behavioral disturbances, such as avoidance behavior [6]. Due to the typically long-lasting duration of the symptoms experienced by affected individuals, anxiety disorders represent more chronic-recurrent than an episodic disorder [7].

Like all psychiatric disorders, anxiety disorders are diagnosed not on objective biomarkers, but based on symptom lists, which refer to a single diagnosis, while patients commonly present symptoms that fit multiple diagnoses [2]. The heterogeneous nature of the population of anxious patients does not only impede diagnosis and discovery of the underlying etiological mechanisms [8], but also contributes to the poor treatment response experienced in many patients [9,10]. Although several established psychotherapeutic and medication-based treatments exist which are effective on average [11,12,13], individual responses to treatments vary widely [9,10], limiting the validity of assumptions that there is a single biological disturbance underlying anxiety in all patients [14,15]. Therefore, disturbances more likely differ among individuals [16], thus requiring the identification of a broader array of biomarkers to gain better insights into patient-specific etiological mechanisms that lead to more targeted treatments [17].

The inclusion of biomarkers that could help improve diagnosis might also help to generate a classification system for psychiatric disorders that can be associated with the dysfunctional pathways underpinning them, finally enabling more targeted treatments of anxiety disorders [16,18]. Among potential biomarkers, the study of metabolites (metabolomics) in a large-scale manner is currently regarded as one of the most informative representations of biological functions, as these molecules carry out or respond to most processes of the body [19,20]. More detailed information on the use of metabolomics in the study of psychiatric disorders is summarized in the following chapter.

## 2. Metabolomics in Studies in Psychiatry

Recent advances in analytical technology enable the so-called “omics technologies”, referring to bioinformatics studies on genes, transcripts, proteins, and metabolites [21,22]. Among these technologies, the study of the metabolome represents the “ome”, which is closest to the phenotype [23]. The word “metabolome” refers to the total metabolite pool in a cell, tissue, or organism [24]. As such, the metabolome consists of a diverse array of biomolecules that are the final products of interactions between gene expression, protein function, and the cellular environment [25]. Metabolites represent the final products and by-products of complex biosynthetic and catabolism pathways. Thus, the study of metabolites in a large-scale manner represents a powerful technique to elaborate phenotypic changes caused by exogenous stimuli more predictively than other omics technologies [24,25,26].

Metabolomics aims to provide detailed and mechanistic insights into the pathology of diseases by revealing altered metabolic pathways. As such, metabolomics is considered to hold potential for the identification of pathways involved in the pathophysiology of diseases and for the diagnosis of psychiatric illnesses [27,28]. Moreover, it offers new options for stratification of patients in a heterogeneous patient population, for monitoring therapeutic efficacy and disease progression, and for defining therapeutic targets [27,28]. All of these aspects have a particular value in complex pathological states, such as psychiatric disorders, as almost all of them are multifactorial conditions, not involving a single-gene mutation [29]. To sum up, metabolomics presents a tool to explore the mechanisms of diseases from a holistic perspective [29]. Therefore, metabolite profiling seems promising to recognize early biochemical changes in disease and, thus, provides an opportunity to develop predictive biomarkers that can initiate earlier interventions [30,31]. The latest applications of metabolomics cover various areas, including screening and diagnostic approaches, discovery and development of new therapeutics, evaluation of drug toxicity and assessment of therapeutic efficacy, patient stratification, and monitoring of patient response to treatment [19,30,31]. Thus, in the future, metabolomics might help to reveal the biological bases of psychiatric symptoms and implement personalized care to patients with mental disorders [32].

Studies based on metabolomic approaches attempt to ascertain biomarkers for diagnosis, disease progression, and the treatment response. Advanced metabolomic platforms enable a global and integrated evaluation of biochemical pathways and metabolic changes appearing in a diseased state [20]. In this regard, the most relevant biological material for the in vivo study of the pathogenesis of psychiatric disorders arguably derives from the brain [33]. Brain tissues, as well as cerebrospinal fluid —which also reflects the metabolic status and biochemistry of the brain—are the most relevant sampling substrates for identifying biomarkers of psychiatric diseases. These brain-derived samples enable the study of causal links between a detected psychiatric pathology and affected molecular pathways. However, such samples from humans are typically only available for analysis at autopsy [20]. Therefore, often animal models are used as tools that help to understand the pathogenesis of psychiatric disorders, as recently reviewed [34]. In humans, however, the use of plasma, serum, or urine has increased in the metabolomic study of mental disorders, which also provides valuable information about the biological signatures of psychiatric disorders. This corroborates to the whole-body concept of psychiatry, based on the fact that although psychiatric disorders seem to be generated in the brain, the effects of these illnesses can be observed throughout the body, as the brain is integrated into virtually all physiological functions of the whole body [20].

Metabolomic studies have already been reported for several psychiatric disorders, including depressive disorders, bipolar disorder, schizophrenia, and drug addiction [19,35]. However, fewer studies have been carried out in anxiety disorders. The following chapters summarize studies on the use of metabolomic platforms to reveal abnormalities and metabolic changes occurring in anxiety disorders. In addition, studies on changes in metabolites due to the treatment of anxiety disorders are summarized. To give a broader overview on changes in blood metabolites, the review is not only limited to advanced metabolomic platforms but also considers studies investigating a smaller set of metabolites by classical methods, such as photometric assays, high-performance liquid chromatography, or gas chromatography.

Therefore, a literature search was conducted using Scopus, PubMed, and APA Psycinfo databases. Research articles in scientific journals on experiments using animal models or human subjects were considered. The search was conducted on 29 May 2020, with no limitations on the publication date. Articles were identified by searching for titles using the following search terms: “(metabolom* OR metabolite OR lipidom OR lipid* OR biomarker) AND (anxi*)”. The search returned 170 records after duplicates were removed.

## 3. Metabolomics to Differentiate Healthy and Anxiety Subjects

As the diagnosis of anxiety disorders still relies rather on symptom checklists than on empirical objective laboratory analyses, efforts have been made to differentiate healthy from anxious subjects by the analysis of metabolites as summarized in Table 1. Anxiety disorders are complex conditions. Numerous factors, including genetic, neurobiological, neurochemical, and psychological factors, are thought to be involved in their development [3]. To elucidate the pathways affected by anxiety disorders, and to identify possible biomarkers, animal studies using brain tissue were conducted. For more detailed information regarding the design of animal studies to serve as models for human anxiety disorders, we refer to our previous review article [34]. In brief, oxidative stress, alterations in lipid and energy metabolism (i.e., mitochondrial regulation), glutamine metabolism, and neurotransmission [36] seem to be involved in anxiety disorders. This overlaps with depressive disorders—which often occur comorbid in individuals with anxiety disorders—where changes in the glutamate–glutamine cycle, as well as changes in lipid and energy metabolism, have also been found to be related to the pathogenesis of major depressive disorder [30].

In human studies, mainly plasma sNamples were used for the study of metabolites. Overall, many early anxiety metabolomics studies focused on lipids (lipidomics), as there is a known connection between lipids and neuronal signaling and disease [45].

Negative correlations between anxiety and high-density lipoprotein (HDL) levels were observed, while higher triglyceride levels were observed in patients with depression and comorbid anxiety compared to depressive patients without anxiety [46]. Furthermore, serum triglycerides, very-low-density lipoprotein (VLDL)-cholesterol and free-cholesterol were higher in patients with anxiety disorders as compared to healthy controls, whereas the opposite was observed for esterified cholesterol [47]. A study conducted in menopausal women observed no correlation between lipid profiles (total cholesterol, HDL, VLDL, low-density lipoproteins (LDL), triglycerides) and anxiety [48]. In young women, on the other hand, low lipid and lipoprotein levels (cholesterol, LDL, total cholesterol, ratio of total cholesterol to HDL) were inversely correlated with anxiety scores [49]. Huang et al. [50] observed differences in HDL cholesterol and the ratio of total cholesterol to HDL with regard to an anxious state in men. In healthy men, levels of total cholesterol and LDL cholesterol were higher in those who scored higher on an anxiety inventory [51]. Thus, several studies support the role of lipids in anxiety disorders, although differences with respect to gender and hormonal status likely exist.

Increasing evidence suggests a crucial role for membrane lipids and lipid oxidation in the pathogenesis of anxiety disorders. Membrane lipids play a pivotal role in the barrier and signaling function of membranes [52]. As dysfunctions in neuronal proteins and peptide activities are considered as a primary cause of anxiety disorders, brain lipids are essential for transmitter signaling. Lipids essential for membrane formation, i.e., n-3 polyunsaturated fatty acids, phospholipids, glycerolipids, and sphingolipids, are assumed to be involved in the pathogenesis of anxiety disorders, especially [53]. The lipid composition of neuronal membranes is highly dynamic and likely affects the assembly of signaling proteins and, thus, neuronal signaling and function [54].

Omega-3 fatty acids serve as precursors for the synthesis of eicosanoids, which might induce perturbations of the system of inflammatory mediators. Anxiety disorders have been linked to inflammation. Thus, the consumption of specific fatty acids or leukotriene receptor antagonists might also contribute to the maintenance of the anxiety symptoms [55].

Given the ubiquitous distribution of lipids at synapsis in the brain, membrane-forming lipids are believed to have high potential in the treatment of anxiety disorders [53]. As such, lipid-based therapies might offer new individualized treatment approaches, such as targeted dietary supplementation of n-3 polyunsaturated fatty acids [56]. Another mode of function might be pharmacological interference of lipids, i.e., glycerolipids, with lipid-regulating enzymes [57].

Observed changes in phospho- and sphingolipids related to anxiety symptoms pinpoint overactive ether lipid cleavage/turnover in the brain in the etiology of anxiety disorders, which likely relate to inflammatory processes [41].

The hypothesis of the association of anxiety with systemic inflammation corroborates a recent finding, showing an association of the inflammation marker C-reactive protein (CRP) [58], with increased risk of suicide in patients with anxiety disorders [59]. Thus, metabolites indicative of poor metabolic health might serve as distal biomarkers for anxiety. Indeed, metabolic health, as indicated by the analysis of 36 biomarkers (e.g., leptin, brain-derived neurotrophic factor, tryptophan), which have been shown to be related to anxiety disorders, revealed the highest occurrence of this mental disorder in individuals with poor metabolic health (the so-called “overweight” class). Therefore, metabolites indicative of poor metabolic health might serve as distal biomarkers for anxiety [60]. However, contrasting results on the association between inflammation and anxiety disorders have been reported. In elderly participants, for instance, a number of systemic inflammation markers (e.g., CRP, interleukins, serum amyloid A, tumor-necrosis factor alpha) were not associated with anxiety symptoms [61]. In another study with apparently healthy women, high-sensitivity CRP and fibrinogen contents were negatively associated with anxiety, whereas no association was observed in men [62]. Therefore, associations of anxiety and micro-inflammation markers also seem to differ with regard to gender and age, which might also contribute to the equivocal results regarding the association of lipid metabolism and inflammation with anxiety symptoms.

Studies also indicate a role of nitro-oxidative stress driving lipid oxidation and lowered lipid-antioxidant defenses in anxiety disorders. More specifically, increased superoxide dismutase, lipid hydroperoxides, nitric oxide metabolites (NOx), and uric acid were measured in individuals with general anxiety disorders than in those without anxiety disorders. Those changes were accompanied by a decrease in HDL and paraoxonase-1 [63]. It is suggested that the inflammation due to the overproduction of NOx is involved in the pathology of anxiety disorders [64]. However, while studies focusing on NOx levels in acute stress models observed associations between anxiety and NOx [65], a study analyzing salivary NOx in daily psychological stress in humans and anxiety observed only correlations between stress and anxiety, but not between salivary NOx and anxiety [64].

Several studies in animals and humans have demonstrated a potential link of anxiety disorders with oxidative stress and lipid peroxidation, as neurochemical causes of anxiety disorders. Lipid peroxidation was enhanced in children with anxiety disorders as compared to a control group, as indicated by increased serum levels of lipid hydroperoxide. Thus, lipid hydroperoxide has been speculated as a potential biomarker for anxiety disorders [3]. Oxidative stress as indicated by elevated levels of lipid hydroperoxide and lower paraoxonase activity (an HDL associated enzyme protecting lipids from peroxidation [66]) have been observed in individuals with generalized anxiety disorder (GAD) without any comorbid psychiatric disorder [67], further supporting the role of lipid peroxidation and oxidative stress in the etiopathogenesis of GAD. Thus, lipid hydroperoxide has been speculated as a potential biomarker for anxiety disorders [3,67].

The association between anxiety and oxidative stress has often been related to nutritional effects. However, other factors might also serve as a source of oxidative stress, such as mobile phone electromagnetic field radiation, vibration and ringtone, which have been found to induce oxidative stress and anxiety-like behavior in rats [68].

As many studies highlight the association between stress and anxiety disorders, salivary cortisone was suggested not only as a stress biomarker but also as a marker of state anxiety [69]. Salivary alpha-amylase—a maker of sympathetic nervous system activity [70]—was observed to be higher in adults with a higher dental anxiety score, thus showing potential to serve as a biomarker of dental anxiety [71]. However, a study conducted in children with and without temporomandibular disorders observed higher anxiety symptoms in children with the disorder, but no difference in salivary alpha-amylase and also salivary cortisol [72]. However, elevated hair cortisol was found to predict later development of anxious behavior in response to a major life stressor in infant monkeys, thus showing some potential as a biomarker for stress-related mental problems [73]. In healthy volunteers exposed to a psychosocial stressor, the anxiety score was associated with salivary alpha-amylase, but not to salivary cortisol or chromogranin-A [74]. Therefore, further studies are needed to clarify whether cortisol, cortisone, and alpha-amylase show potential as biomarkers for anxiety disorders.

The neuropeptide pituitary adenylate cyclase-activating polypeptide (PACAP) is assumed to be involved in stress response and has been suggested as a biomarker for the severity of stress-related psychiatric disorders [75]. Serum PACAP analysis in male and female individuals diagnosed with GAD compared to healthy controls revealed no overall association between circulating PACAP and GAD, but an association in women [76], supporting prior work suggesting potential sex differences in PACAP effects, likely due to estrogen-dependent regulation of this pathway [75].

The neurotrophin fibroblast growth factor-2 (FGF2)—a protein involved in stress regulation and neurogeneration [77]—is also considered as an endogenous regulator of fear expression. Thus, FGF2 might also serve as a potential biomarker for anxiety disorders [78]; however, further research is required to elucidate the potential of FGF2 to identify vulnerable individuals and to establish preventative interventions.

Studies also aimed to integrate biopsychosocial aspects of stress, immune markers, and behavior in the development of anxiety symptoms. Chronic stress causes perturbations in the hypothalamus–pituitary–adrenal (HPA)-axis, which might mediate the relationship between cardiovascular diseases and affective disorders [79]. One study investigated relations between stress, HPA-axis, and mother–child interaction patterns on the development of anxiety in children exposed to chronic trauma [80]. Trauma-exposed children exhibited more anxiety symptoms, which might be explained by three bio-behavioral paths: a mediated biological pathway through HPA-axis functioning (higher salivary cortisol in trauma-exposed mothers and also children), another biological pathway via the immune system (higher salivary immunoglobulin A (IgA) in trauma-exposed mothers and also children), and a third path with a behavioral link from diminished maternal supply to exposure to child anxiety. Moreover, anxiety in children exposed to continuous wartime trauma integrating endocrine and behavioral measures from mother and child was researched previously [81]. The study revealed that maternal physiology and behavior impacted child anxiety and three possible pathways were defined: augmentation of child anxiety through increased maternal salivary IgA, which led to enhanced child IgA; reduced social repertoire of the child due to reduced maternal oxytocin—and, in turn, reduced child oxytocin; and a direct impact of increased maternal anxiety on child anxiety.

Previous studies also attempted to reveal biological aspects of the higher prevalence of anxiety disorders in women. Differences in the hormonal status, i.e., with respect to the steroid pattern, have been speculated to be a reason behind. Higher levels of estrogens in women with anxiety disorders, when compared to women with depression, have been observed [82].

In one study, a specific analysis of the steroid metabolome in the blood of men with anxiety or depression compared to healthy controls was carried out. Conjugated steroid forms, i.e., sulfates, such as pregnenolone sulfate, differed between all three groups, and, thus, also provide an opportunity to serve as biomarkers to differentiate depressed from anxious individuals [83]. Among the previously considered steroids as being neuroactive, steroid sulfates, such as pregnenolone sulfate, are reported to act as negative gamma-aminobuytric acid (GABA) receptor modulators [84], which might explain the lower pregnenolone sulfate concentration in anxious and depressive men.

Besides plasma analysis, metabolomics analyses were also conducted on urine samples. Zheng et al. [44] used different metabolomics approaches to profile urine samples from healthy controls and patients with depression and anxiety disorders. Overall, four biomarkers—*N*-methylnicotinamide, aminomalonic acid, azelaic acid, and hippuric acid—were identified as being able to distinguish healthy from depressed/anxious individuals. Those biomarkers were mainly involved in three metabolic pathways (tryptophan–nicotinic acid metabolism, lipid metabolism, tyrosine–phenylalanine pathways) and five molecular and cellular functions (cell cycle, amino acid metabolism, molecular transport, cellular growth and proliferation, small molecule biochemistry).

Further specific studies investigated comorbid anxiety disorders in specific disorders, such as autism, cancer, complex regional pain syndrome, or Cushing’s syndrome. Only a few recent studies related to these specific diseases are summarized below.

Central and peripheral metabolites in patients with complex regional pain syndrome were analyzed for their association with psychological disorders, including anxiety [85]. Specific associations were observed, which might show pathological interactions between a painful body and increases in anxiety in this population. Strong positive correlations between valine/*N*-acetylaspartylglutamate (val/tNAA) and anxiety in the right thalamus were observed. Lower NAA levels have been related to dysfunctional cell death related to neurons and glia cells. As lower NAA levels have been observed in patients with complex regional pain syndrome before, neuronal cell death may affect anxiety symptoms in this population [86,87]. In addition, peripheral CO_2_ was positively associated with anxiety, which might be explained by an increase in sensory pain levels due to increased partial CO_2_ pressure causing a synergistic boost of neuropsychiatric symptoms, such as anxiety [85].

An investigation in individuals with Cushing’s syndrome evaluated the deleterious effects of excessive glucocorticoid exposure on neuronal changes related to anxiety [88]. Metabolomic analyses revealed a negative correlation of *N*-Acetyl-aspartate (a marker of neuronal integrity and viability [89]) and creatinine (a marker for brain cell density in glial and neuronal cells, and energetic systems [90]) with anxiety, suggesting that long-term exposure to excessive glucocorticoid levels causes metabolic alterations in the prefrontal cortex associated with anxiety [88].

A study in patients with colorectal cancer undergoing three different stages of therapy, observed clinically relevant anxiety and/or depression levels in all patients [91]. Serum levels of fractaline (a chemokine involved in the progression of different types of tumors [92] and also in the inhibition of neurotransmission related to anxiety [93]) were positively correlated with anxiety scores. Therefore, fractaline might serve as a biomarker for the detection of anxiety disorders in cancer patients, and they might also assist in the development of personalized anxiolytic treatment strategies for cancer patients [91].

## 4. Metabolomics in the Study of the Role of the Gut Microbiome in Anxiety Disorders

The gut microbiome is suggested to play a pivotal role in the induction of anxiety-like behavior, through stress-induced dysbiosis [4]. The link of the gut microbiome and stress-related conditions has largely been investigated in studies with germ-free animals. A recent study in rats subjected to chronic unpredictable stress revealed that changes in the gut microbiome were accompanied by dysregulation of plasma metabolites related to metabolism of glycerophospholipids, glycerolipids, fatty acyls, and sterols [94]. The authors suggest that lactate produced from gut microbes might possibly promote anxiety-like behavior through the modulation of fatty acid metabolic pathways, resulting in low levels of plasma fatty acids. It was suggested that the future development of treatment strategies for anxiety disorders should consider targeting sphingolipid receptors.

In a further study with germ-free animals, these subjects had higher serotonin metabolite levels compared to conventionally raised controls. It was also suggested that the gut microbiome can affect the serotonergic neurotransmission in the central nervous system, through a humoral route, based on the finding of higher concentrations of the serotonin-precursor tryptophan in the plasma of germfree animals [95].

Furthermore, gender-differences in anxiety disorders have been observed in animal studies [4]. For instance, dietary supplementation with the n-3 polyunsaturated fatty acid docosahexaenoic acid (DHA) in male socially isolated mice reduced anxiety behaviors compared to controls, whereas no differences occurred in female mice [96]. In addition, a sex-specific interaction of the DHA-supplementation with the gut microbiome was observed, showing a significant effect on the microbiome in male but not in female mice.

Besides animal studies, human studies with respect to the role of the gut microbiome were also conducted, which mainly relied on correlative analysis. In this regard, Stevens et al. [97] investigated fecal microbiota in humans with anxiety or depressive disorders as compared to control reference subjects. Gut dysbiosis in anxious and depressed individuals and over-representation of lipopolysaccharide (LPS) biosynthesis genes in the gut microbiome were reflected in changes in metabolic pathways of mood neurotransmitters as well as deleterious metabolism of intestinal protective mucin and elevation of plasma LPS, and epithelium integrity molecules. These results support the notion that LPS might compromise the integrity of the gut barrier, causing systemic manifestations, including the brain [98].

The microbiota–gut–brain axis is also assumed to play a central role in the etiology of depression, showing that disturbances in the gut microbiome disturb metabolic homeostasis [30]. Several studies provide support that dysregulation of the enteric microbiome does not only produce detrimental metabolites but also causes increased bacterial translocation across the intestinal tract. These processes are assumed to be involved in the pathophysiology of anxiety and depressive disorders through proinflammatory cytokines and neuroinflammation, the HPA-axis, and vagal nerve activation, as reviewed recently [94]. Overall, based on these studies, future studies should elucidate the role of the gut as a novel target for the treatment of anxiety disorders.

## 5. Metabolomics in the Study of the Role of Nutrition in Anxiety Disorders

Several animal studies were conducted to evaluate the impact of different diets on anxiety. Some studies investigated the effect of maternal diet on the offspring. For instance, one study investigated the effect of maternal consumption of conjugated linoleic acid during gestation and lactation on cerebral lipid peroxidation and anxiety behavior in rats [99]. Higher levels of the antioxidant glutathione together with a lower concentration of the lipid peroxidation marker malondialdehyde were observed in brain tissues in the offspring of rats receiving conjugated linoleic acid. Maternal intake of conjugated linoleic acid also caused an anxiolytic effect in the offspring. Therefore, results imply that an adequate supply of essential fatty acids during pregnancy plays an important role in facilitating the development of the nervous system and protecting the offspring from neuronal changes, such as those leading to anxiety.

In a further study, the offspring of rats received a diet consisting of high contents of simple carbohydrates, saturated or trans-fats, sodium, and low protein and fiber contents (a so-called “cafeteria diet”) during lactation and/or post-lactation compared to rats receiving a control diet [100]. The effects of this cafeteria diet on physiological parameters and anxiety were investigated. The highest triglyceride levels were found in the offspring of rats receiving post-lactation cafeteria diet or total cafeteria diet. The offspring also presented higher levels of anxiety compared to the control groups and groups with only a lactational cafeteria diet. Thus, the study provides some evidence that the ingestion of a cafeteria diet after lactation might trigger metabolic (increase in serum triglycerides and oxidative stress) and behavioral alterations (anxiogenic effects) in rats.

A broad range of studies investigated nutritional biomarkers and anxiety during pregnancy and postpartum in humans, as recently systematically reviewed by Trujillo et al. [101]. Most relevant studies are briefly described in the following.

One study related to anxiety disorders during pregnancy to nutritional biomarkers. Associations between polyunsaturated fatty acids and anxiety disorders in early pregnancy were observed, showing an inverse relation of serum DHA levels and anxiety disorders in the first trimester [102]. Furthermore, associations between cholesterol and anxiety in the postpartum period were investigated, as total lipids decrease considerably after delivery as compared to pregnancy [103]. Overall, only moderate negative associations between total cholesterol and HDL cholesterol and anxiety symptoms were observed in the postpartum period [104].

Next to the effects of fatty acids, a possible association between amino acids and anxiety was also studied [105], showing an inverse relationship of the ratio of plasma tryptophan and the sum of the levels of valine, leucine, isoleucine, and phenylalanine with anxiety. Moreover, changes in plasma phenylalanine were correlated with changes in anxiety scores from the 3rd to 6th day before delivery to the 1st and 3rd postnatal day; however, these associations should be interpreted with caution, as only low correlations (r = 0.16, *p* = 0.04) were observed [106].

The role of micronutrients has hardly been investigated so far, showing no association of vitamin D with anxiety in pregnancy [107] and also no correlation between zinc levels and anxiety during pregnancy and in the postpartum period [108].

Further studies investigated possible associations between obesity/metabolic syndrome and anxiety disorders using animal models. For instance, in rats fed a high-saturated fat or a high-fat and high-fructose diet, behavioral alterations toward anxiety-like behavior were observed [109]. These behavioral alterations correlated with dyslipidemia (increased serum triglycerides and cholesterol), lipid peroxidation, and metabolic parameters. Long-term feeding of high-fat diets has also shown to increase malondialdehyde concentrations and to decrease glutathione levels in the serum of rats, which went along with increases in anxiety-like behavior [110].

In humans, a cross-sectional study investigated associations of anxiety and metabolic syndrome components in metabolic syndrome patients. Waist, body mass index, and degree of obesity, and the hypertension component could be linked to systolic blood pressure, pulse pressure, total cholesterol, and trait anxiety, but not to state anxiety. Thus, cholesterol metabolism, blood pressure, and high trait-anxiety likely interact in the pathophysiology of hypertension in metabolic syndrome [76].

Studies conducted in animals and humans reveal an inverse relationship of the dietary total antioxidant capacity with oxidative stress biomarkers as well anxiety [111]. Therefore, lipid peroxidation does not only seem to play a role during pregnancy/early life, but also in adults.

Overall, research indicates that nutrition—mainly associated with lipid peroxidation, inflammation, and metabolic alterations—plays a role in translating diet-induced metabolic alterations into anxiety disorders. Therefore, fatty acids, such as n-3 polyunsaturated fatty acids, or the provision of antioxidants, are also considered as new treatment options [53,110,111].

## 6. Metabolomics in the Study of Anxiolytic Effects

Metabolomics was also applied in the field of drug discovery, including natural product research.

Several studies used metabolomics analyses to characterize the composition of anxiolytic drugs/natural products [112], which will not be described in more detail as it does not fall within the scope of this review. However, in some studies, changes in brain or plasma metabolites due to drug administration were also assessed, as summarized in Table 2. These studies highlight the role of the effects of anxiolytic drugs on neurotransmitter metabolism, but also on antioxidant mechanisms. Several studies pinpoint the involvement of changes in serotonergic activity in the anxiolytic effect of several drugs, showing increasing serotonin contents in rodent brains [113,114]. In addition, the role of dopamine in anxiety has been reported before, revealing increasing concentrations in the prefrontal cortex during stressful and anxiogenic situations [113], and a decrease after the administration of anxiolytic drugs, such as afobazole [114]. However, for other drugs, such as diazepam, no effect on the dopamine content was observed, despite their anxiolytic activity [113]. Next to serotonin and dopamine, the glutamate–glutamine cycle in the brain plays an essential role in mental disorders [115], as glutamate represents the primary and most abundant excitatory neurotransmitter in the central nervous system. The functionality of the glutamate–glutamine cycle is essential for glutaminergic neurotransmission [116]. Furthermore, glutamine is not only essential as a precursor for the neurotransmitter glutamate but also for the neurotransmitter GABA [115] and the antioxidant glutathione [117]. The dysfunction of the glutamate–glutamine cycle is suggested to be involved in different forms of anxieties [40]. Therefore, several anxiolytic drugs might pose their anxiolytic effects via their impact on this cycle, as summarized in Table 2.

In the following, only a few recent studies using metabolomic approaches in the study of anxiolytic effects are reported in more detail.

The ethanol extract of Passiflora edulis Sims F. flavicarpa was tested in comparison to a positive drug control (diazepam) in a randomized trial using an anxiety model in rats. Administration of *P. edulis* extract enhanced GABA concentrations in the brain and exhibited an anxiolytic-like effect. Thus, it is assumed that *P. edulis* extracts might function as positive allosteric modulators of GABA. Using metabolomics approaches, secondary metabolites were investigated and correlated with measured activities. However, no correlation of the different metabolites was observed, suggesting that the anxiolytic effect is not attributable to a single metabolite, but rather to an additive or synergistic effect of several entities [118].

In line with the research indicating a role of oxidative stress in the etiology of anxiety disorders, a meta-analysis by Aponso et al. [119] reported anxiolytic effects of inhaled essential oils as well reduced oxidative stress. Moreover, extracts of Hypericum Scabrum—a phyto drug with antioxidant properties—were shown to be able to reverse diet-induced alterations related to oxidative stress [110]. More specifically, detrimental effects of high levels of saturated fats on oxidative status and anxious behavior were observed in rats. A long-term high-fat diet enhanced serum malondialdehyde levels, decreased glutathione levels, and enhanced anxiety. The extract of H. scabrum inversed these diet-induced alterations and decreased anxiolytic effects. Therefore, it is expected that phytomedical, natural therapeutic agents with antioxidant properties might offer preventative and/or curative measures in anxiety disorders.

The linkage of psychological stress and production of free radicals with anxiety disorders was also used to investigate oxidative metabolites as biomarkers for monitoring the response to treatment with anxiolytics in a randomized placebo-controlled study [120]. Biopyrrins, the oxidative metabolites of bilirubin, were investigated in urinary samples of mice receiving the anxiolytic alprazolam subjected to acute stress. In addition, corticosterone levels in serum were analyzed. An increase in biopyrrins in stressed mice and a decrease after the anxiolytic treatment, as well as a correlation between urinary biopyrrins and serum corticosterone levels, were observed, thus showing some potential for urinary biopyrrins to serve as biomarkers for the assessment of the response to anxiolytics.

Indicators of stress and lipid peroxidation were also investigated in the brain of psychologically-stressed mice receiving anxiolytic and anxiogenic drugs [121]. The content of thiobarbituric acid reactive substances—an index of lipid peroxidation activity—was enhanced in the brain, but not in the liver or serum after stress exposure. The oxidative brain damage in the brain lipids went along with the enhanced production of nitric oxidase through the mediation of non-selective nitric oxidase synthase. The stress-induced detrimental effects were suppressed by anxiolytic drugs. Thus, drugs with benzodiazepine or a serotonin receptor agonist profile might pose anxiolytic effects due to their protective effects on stress-induced oxidative brain damage.

Further studies point at the pivotal role of the antioxidant effects of anxiolytics. For instance, the anxiolytic-like effect and the possible neuronal mechanism of action of the chemical isopentyl ferulate (IF) were investigated in a randomized trial in mice with a negative control group. Overall, the calming effect of IF went along with a decrease in hippocampal nitrite and lipid peroxidation levels and an increase in glutathione and antioxidative enzymes (glutathione peroxidase, superoxide dismutase, catalase). Further investigations regarding possible involvement of the GABAergic system in the anxiolytic effect of IF yielded some evidence that IF might show neuroprotective effects through the GABAergic transmission pathway [122].

Anxiolytic effects of satins—drugs that are used to lower LDL levels—were also discussed in a recent review article [123]. The mechanisms behind it are assumed due to a modulation of the *N*-methyl-d-aspartate (NMDA) receptors in the brain, which show a close correlation with anxiety-like behavior. Statins can disable these NMDA receptors due to their role in the disruption of membrane/lipid rafts, finally disabling the NMDA receptor-mediated anxiety.

In a case study in a patient with a treatment-refractory substance use disorder and comorbid anxiety and depressive symptoms, repeated transcranial magnetic stimulation was successful in reducing anxiety symptoms [126]. It was speculated that enhanced glutamate transmission in the corticostriatal pathways occurred due to the stimulation of the dorsolateral prefrontal cortex. This might, in turn, modulate the GABA/glutamate balance within the basal ganglia, which, in turn, promotes dopamine release in the mesocortical pathways, finally reducing psychiatric symptoms.

Lifestyle changes, such as nutritional changes or exercise, have been proposed as possible complementary modalities to prevent and cure disorders, and the combination of both approaches, i.e., dietary supplementation with polyunsaturated fatty acids in combination with physical exercise, showed synergistic effects on brain function and behavior [127,128]. A study in mice investigated the effect of voluntary running on anxiety-like behavior and the lipid metabolome in the brain and blood corticosterone levels. Compared to sedentary mice, the running group displayed lower anxiety-like behavior, which went along with differences in blood corticosterone and a region-specific cortical decrease in the palmitate (C16:0) and a concomitant increase in arachidonic acid and DHA. Therefore, it is assumed that the anxiolytic effects of physical exercise derive from exercise-induced activation of cortical signaling cascades involving or dependent on bioactive lipids [129]. In humans, physical exercise (strength and endurance training) reduced anxiety, which went along with a reduction in CRP, an indicator of cardiac veins inflammation [130], with the latter being stronger affected by strength than endurance training [131]. *Tai Chi Chuan* is often viewed by Chinese people as physical exercise to improve mind–body health, therefore, making it an interesting research target in the field of cardiovascular health and anxiety symptoms. Thus, a randomized-controlled trial was conducted to evaluate the effect of a *Tai Chi Chuan* exercise program on anxiety status and blood lipid profile in individuals with hypertension as compared to healthy subjects [132]. As an increase in HDL and a decrease in total cholesterol, LDL, and triglycerides went along with decreases in trait and state anxiety, it was suggested that *Tai Chi* might be used as an alternative treatment in patients with anxiety disorders.

## 7. Conclusions

The aforementioned studies on the use of metabolomics in anxiety disorders are promising for diagnosis, gaining insight into the etiology of the disorders and the development of treatment strategies. Overall, metabolites related to oxidative stress, inflammatory processes, lipid and energy metabolism, glutamine metabolism, and neurotransmission seem to pose the potential to serve as biomarkers for anxiety disorders; however, to date, the application in clinical practice is not feasible due to several limitations. The main limitation is that, so far, no references for normal ranges of metabolites exist [30]. Furthermore, variables, such as gender, diet, or lifestyle, affect the metabolic profile and also medical comorbidities and the use of medications or drugs need to be considered and, thus require further research [133,134]. Furthermore, many findings of this systematic review are based on animal studies. Those studies on human anxiety were also prevalent beneath other disorders. It must be taken into account that the group of anxiety disorders is from the clinical and etiological point of view very different. Thus, a more specific approach, according to the different categories of anxiety disorders, might be more efficient. There is also a lack of research on whether metabolomic biomarkers can predict or moderate treatment response to anxiolytic medication and psychotherapy. However, in major depressive disorders, for instance, studies indicate predictive potential of the pretreatment metabolomics profile of the response to antidepressant medication [135], and also one pilot psychotherapy study revealed that several plasma metabolites might serve as moderators of the outcome of psychotherapy [136]. Future studies should also explore metabolomics changes in anxiety due to psychotherapy treatment, which might also help to understand better the mechanistic underpinnings of the effect of psychotherapy on symptom change and whether these changes are associated with metabolomics alterations. Therefore, more research is needed to reveal whether metabolomics can provide biomarkers to improve treatment selection and personalized treatment for patients with anxiety disorders.

## Figures and Tables

**Table 1:** Possible biomarkers identified to differentiate healthy and anxiety subjects.

Subject	Sampling Material	Analytical Platform	Metabolites Identified	Pathways Involved/Functions	Reference
Mice	Plasma	GC-MS ^1^	Myo-inositol, glutamate, tricarboxylic cycle-intermediates	Mitochondrial energy pathways, inositol pathways, HPA ^2^-axis, glutamate metabolism	[37]
Mice	Brain	GC-MS	Dehydroascorbate, xylose, succinic acid	Energy metabolism, mitochondrial import and transport, oxidative stress, neurotransmission	[38]
Mice	Brain and plasma	LC-MS/MS ^3^	1-methyl histidine, deoxyuridine, kynurenic acid, 2-hydroxygluterate, carnitine, acetylcarnitine, cytosine	Oxidative stress, energy metabolism, amino acid metabolism neurotransmitter metabolism	[39]
Dogs	Plasma	LC-MS ^4^	Glutamine, γ-glutamyl–glutamine	Glutamine metabolism	[40]
Humans	Plasma	LC-MS/MS	Phosphatidyl-cholines (PC O 36:4), ceramides (CER 20:0)	Phospho- and sphingolipid metabolism	[41]
Humans	Plasma	Photometric assays, immuno-assays	Cholesterol (HDL ^5^, LDL ^6^), fructosamine, triglycerides, free fatty acids, dehydro-epiandrosterone-sulfate, adrenocorticotropic hormone	Lipid and carbohydrate metabolism	[42]
Humans	Plasma	not specified	Cholesterol, triglycerides, apolipoproteins B	Lipid metabolism	[43]
Humans	Urine	GC-MS	*N*-methylnicotin-amide, amino-malonic acid, azelaic acid, hippuric acid	Tryptophan–nicotinic acid metabolism, lipid metabolism, tyrosine–phenylalanine pathways	[44]

^1^ GC-MS, gas chromatography–mass spectrometry. ^2^ Hypothalamus–pituitary–adrenal. ^3^ LC-MS/MS, liquid chromatography–tandem mass spectrometry. ^4^ LC-MS, liquid chromatography–mass spectrometry. ^5^ HDL, high-density lipoproteins. ^6^ LDL, low-density lipoproteins.

**Table 2:** Overview of metabolomic studies in the study of anxiolytic effects.

**Subject**	**Sampling Material**	**Analytical Platform**	**Anxiolytic Drug**	**Metabolites Identified**	**Pathways Involved/Functions**	**Reference**
Mice	Brain	NMR ^1^	Specific herbal formula (Fu Fang Jin oral liquid)	ATP, fumarate, malate, lactate, glycine, GABA ^2^, *N*-acetyl-aspartyl-glutamate	Energy metabolism, choline metabolism, neuro-transmitter metabolism	[124]
Mice	Brain	HPLC ^3^	(Z)-3-hexenol, Diazepam	Serotonin (5-hydroxy-tryptamine; 5-HT ^4^), 5-hydroxyindoleacetic acid	Neuro-transmitter metabolism	[113]
Rats	Brain	not specified	Afobazole, Ladasten	3,4-dihydroxy-phenylacetic acid, homovanillic acid, 5-HT, 5 oxytryptophan, 5-hydroxyindoleacetic acid, l-3,4-dihydroxy-phenylalanine	Neuro-transmitter metabolism	[114]
Rats	Brain	HPLC-ED ^5^	Imipramine	5 oxytryptophan, homovanillic acid, dihydroxyphenylacetic acid	Neuro-transmitter metabolism	[125]
Rats	Brain	UPLC-MS ^6^	Passiflora edulis Sims F. flavicarpa, diazepam	GABA	Neuro-transmitter metabolism	[118]
Mice	Urine, serum	Immuno-assays	Alprazolam	Biopyrrins, corticosterone	Oxidative stress	[120]
Mice	Brain	Antioxidant assays	Isopentyl ferulate	Nitrite and lipid peroxidation markers, glutathione, glutathione peroxidase, superoxide dismutase, catalase	Oxidative stress, neuro-transmitter metabolism	[122]

^1^ NMR, nuclear magnetic resonance spectroscopy. ^2^ GABA, gamma-aminobuytric acid. ^3^ HPLC, high-performance liquid chromatography. ^4^ 5-HT, serotonin, 5-hydroxytryptamine. ^5^ HPLC-ED, high-performance liquid chromatography—electrochemical detection. ^6^ UPLC-MS, ultra-performance liquid chromatography—mass spectrometry.

## Abbreviations

CER	Ceramides
CRP	C-reactive protein
DHA	Docosahexaenoic acid
DOPA	Dopamine
FGF2	Fibroblast growth factor-2
GABA	Gamma-aminobuytric acid
GAD	Generalized Anxiety Disorder
GC-MS	Gas chromatography–mass spectrometry
HDL	High-density lipoprotein
HPA	Hypothalamus–pituitary–adrenal
HPLC	High-performance liquid chromatography
HPLC-ED	High-performance liquid chromatography–electrochemical detection
5-HT	Serotonin
IgA	Immunoglobulin A
LC-MS	Liquid chromatography–mass spectrometry
LC-MS/MS	Liquid chromatography–tandem mass spectrometry
LDL	Low-density lipoprotein
LPS	Lipopolysaccharides
NAA	*N*-acetylaspartylglutamate
NMR	Nuclear magnetic resonance spectroscopy
NOx	Nitric oxide metabolites
PACAP	Pituitary adenylate cyclase-activating polypeptide
PC	Phosphatidylcholines
UPLC-MS	Ultra-performance liquid chromatography–mass spectrometry
VLDL	Very low-density lipoprotein
